# Intensive blood pressure control in type 2 diabetes: a meta-analysis of randomized controlled trials

**DOI:** 10.3389/fendo.2026.1850865

**Published:** 2026-06-15

**Authors:** Zeguang Liu, Junfeng Wang, Chenguang Niu

**Affiliations:** 1 Department of Cardiology, The First Affiliated Hospital of Henan University, Kaifeng, China; 2 Central Laboratory, Henan University School of Stomatology, Kaifeng, China

**Keywords:** hypertension, intensive blood pressure control, major adverse cardiovascular events, meta-analysis, type 2 diabetes

## Abstract

**Background:**

Although guidelines recommend lower blood pressure targets for patients with type 2 diabetes (T2D), evidence from randomized controlled trials (RCTs) remains inconsistent regarding the balance between reducing major adverse cardiovascular events (MACE) and the risk of adverse events.

**Methods:**

This meta-analysis included RCTs comparing intensive versus conventional blood pressure control in patients with T2D. The primary outcome was MACE, and secondary outcomes included adverse events. Data were synthesized using a random-effects model. Subgroup analyses were conducted to assess the influence of different blood pressure targets and concomitant intensive glucose or lipid management.

**Results:**

Nine trials comprising 34,260 participants with T2D were included. Intensive blood pressure control was associated with a significantly lower risk of MACE compared with conventional control [7.9% vs 9.7%; risk ratio (RR), 0.80; 95% CI, 0.73–0.89; *P* < 0.001]. Among individual MACE components, intensive treatment significantly lowered the risk of stroke (RR, 0.73; 95% CI, 0.60–0.87; P = 0.008), whereas no significant differences were observed for heart failure, myocardial infarction and cardiovascular death risk. Subgroup analyses suggested a potentially treatment benefit in trials aiming for a systolic blood pressure (SBP) target of <130 mm Hg and incorporating an explicit diastolic blood pressure (DBP) target Concomitant intensive glucose or lipid control did not significantly modify the treatment effect. Regarding safety, intensive treatment was associated with a borderline increased risk of hypotension (RR, 4.61; 95% CI, 1.01–20.99; *P* = 0.05) but not with other adverse events.

**Conclusions:**

Intensive blood pressure control significantly reduces the risk of MACE in patients with T2D. Strategies incorporating explicit DBP targets may offer substantial benefits. However, it should be paid attention to monitoring and preventing adverse events.

**Systematic review registration:**

https://www.crd.york.ac.uk/PROSPERO/, identifier CRD420251266814.

## Introduction

Approximately 10% of adults have type 2 diabetes (T2D), and the risk of cardiovascular disease is more than double that of adults without T2D (1–3). The 2025 ACC/AHA and 2024 ESC guidelines recommend a target systolic blood pressure (SBP) below 130 mm Hg for patients with T2D to reduce the risk of cardiovascular disease (4, 5). These recommendations are primarily based on the cardiovascular benefits of intensive lowering of blood pressure observed in hypertensive populations (6).

However, the evidence in T2D patients remains limited. The BPROAD trial suggested that intensive blood pressure control may reduce cardiovascular disease risk, whereas the ACCORD trial did not show consistent benefits for major adverse cardiovascular events (MACE) and even reported a higher incidence of adverse events (7, 8). Moreover, most trials have focused on MACE, with limited attention given to adverse events such as hypotension and hypokalemia (9, 10). Furthermore, current evidence remains insufficient for various clinical settings, including whether lower SBP targets, explicit diastolic blood pressure (DBP) targets, and concomitant intensive glucose or lipid control provide incremental benefits for patients with T2D (11–13).

To address these uncertainties, we conducted this meta-analysis of randomized controlled trials (RCTs) to assess the association of intensive blood pressure control with MACE and adverse events compared with conventional control in patients with T2D. We also assessed the consistency of treatment effects across different intervention strategies.

## Methods

### Protocol and search strategy

This meta-analysis followed the PRISMA guidelines (**Supplementary Table 1**) and was prospectively registered in PROSPERO (https://www.crd.york.ac.uk/PROSPERO/view/CRD420251266814) (14).

Four electronic databases (PubMed, Embase, the Cochrane Library, and Web of Science) were systematically searched from inception to December 12, 2025. The search strategies combined controlled vocabulary and free-text terms related to diabetes, blood pressure, and treatment intensity (**Supplementary Table 2**). No language restrictions were applied. Additionally, the reference lists of the included studies were manually screened to identify further eligible articles.

### Inclusion and exclusion criteria

Eligible studies included RCTs involving patients with T2D, comparing intensive blood pressure control (SBP ≤130 mm Hg) with conventional blood pressure control (a higher SBP target or a less intensive BP management strategy) and at least reporting MACE. Studies were excluded if full-text reports or outcome data were unavailable.

Two researchers independently screened the literature. Discrepancies were resolved by consensus or consultation with a third reviewer. Titles and abstracts were first screened to identify potentially eligible studies, followed by full-text review for final inclusion.

### Data extraction and quality assessment

Data were extracted from each included trial according to the intention-to-treat principle whenever available, covering the following domains (1): general study characteristics (first author, year of publication, journal, country or region, and study design) (2); clinical and methodological variables (follow-up duration, total sample size, mean age, proportion of male participants, prespecified target blood pressure, intervention strategies, baseline blood pressure, and achieved blood pressure); and (3) outcome data (MACE, heart failure, myocardial infarction, stroke, cardiovascular mortality, hypotension, arrhythmia, syncope, renal impairment, hypokalemia, and hyperkalemia). When specific data were not explicitly reported, values were derived from available reported statistics.

Study quality was assessed using the Revised tool for assessing risk of bias in randomized trials (RoB 2) (15). Two reviewers independently evaluated five domains (randomization process, deviations from the intended interventions, missing outcome data, measurement of the outcome, and selection of the reported result). Each domain was rated as low risk of bias, some concerns, or high risk of bias. Any discrepancies were resolved through discussion or, if necessary, consultation with a third reviewer.

### Statistical analysis

Risk ratios (RRs) with 95% confidence intervals (CIs) were used as the measure of treatment effect. A continuity correction of 0.5 was applied to studies with zero events in either arm. Additionally, sensitivity analyses for sparse data were performed using Mantel-Haenszel methods without continuity correction. Data synthesis was performed using a random-effects model with the Restricted Maximum Likelihood (REML) estimator (16, 17). To account for the uncertainty in the variance estimation, the Hartung-Knapp adjustment was applied to calculate the 95% CIs (18). For outcomes with ≤3 studies, we used standard REML method due to unstable variance estimates. Heterogeneity was evaluated using the Cochran Q test and quantified with the I² statistic. Sensitivity analyses were conducted using a leave-one-out approach to assess the robustness of the findings. Publication bias was assessed by visual inspection of funnel plots and statistically computed using Egger’s regression test.

Subgroup analyses were stratified by blood pressure targets and concomitant metabolic management. Interaction between treatment effect and subgroup variables was assessed using the Q test for heterogeneity between subgroups. Trial Sequential Analysis (TSA) was performed to control for the risks of random errors and repetitive testing (19). The required information size (RIS) was estimated based on a two-sided type I error (α) of 5%, a power of 80%, and an anticipated RR reduction of 20% (7, 8). The variance for the RIS estimation was adjusted for heterogeneity. O’Brien-Fleming α-spending monitoring boundaries were constructed to determine efficacy or futility.

All statistical analyses were performed using Python (version 3.9). The SciPy and Pandas libraries were utilized for statistical modeling and data management, while Matplotlib was used to generate forest plots, funnel plots, and TSA figures. A two-sided *P* value of <0.05 was considered statistically significant.

## Results

### Baseline characteristics of the included studies

**Figure 1:**
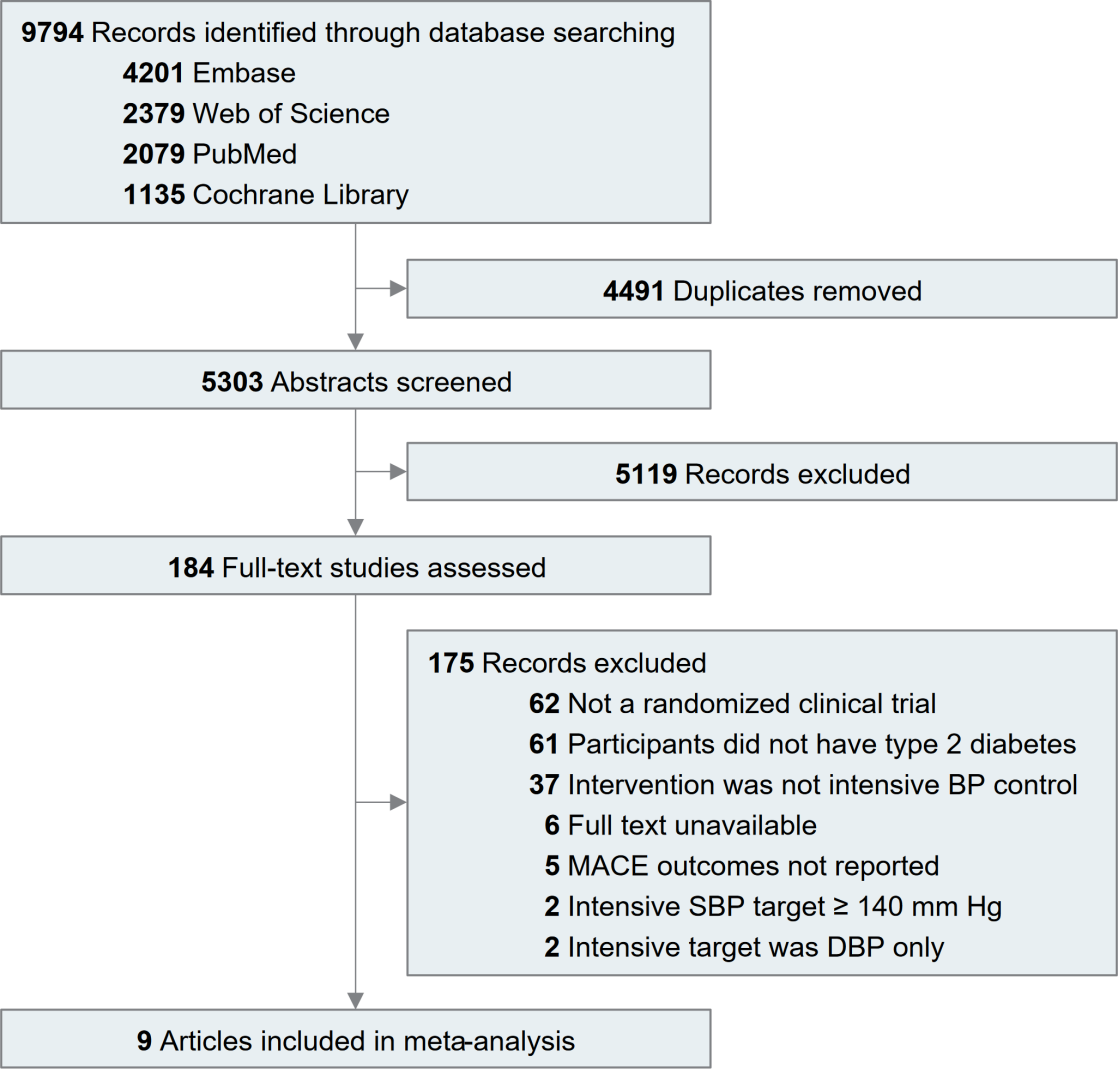
PRISMA flow diagram of study selection. BP indicates blood pressure; DBP, diastolic blood pressure; and SBP, systolic blood pressure.

The initial search across four databases identified 9,794 records. After deduplication and title/abstract screening, 184 records underwent full-text review. Following full-text assessment, nine studies involving a total of 34,260 patients (mean age, 63.3 years; 51% men) with T2D were included in this meta-analysis (**Figure 1**) (7–10, 20–24).

The target of SBP control in these studies generally ranged from 115 to 130 mm Hg in the intensive group (**Table 1**). Six studies focused on patients with T2D (7–10, 21, 23), and three were diabetic subgroups from hypertension studies (20, 22, 24). Five studies employed intensive blood pressure control as the sole intervention (7, 8, 20, 22, 24), whereas the remaining four involved concurrent intensive glucose or lipid control (9, 10, 21, 23). All studies had follow-up durations of at least three years. In the NID-2 trial, both arms shared a blood pressure target of 130/80 mm Hg; the groups were distinguished by the treatment strategy (standardized algorithmic intervention versus flexible outpatient care) rather than different blood pressure targets (23).

**Table 1:** Characteristics of the trials included in the meta-analysis.

Study name	Follow-up, y	Region	Intensive glucose control	Intensive lipid control	Sample size	Mean age, y	Male, %	Intensive BP target, mm Hg
CRHCP (22), 2025	3.0	China	No	No	6,303	63.2	34	130/80
BPROAD (7), 2025	5.0	China	No	No	12,821	63.8	55	120
ESPRIT (20), 2024	3.0	China	No	No	4,359	64.6	59	120
STEP (24), 2023	4.0	China	No	No	2,274	66.4	45	130
NID-2 (23), 2021	10.5	Italy	Yes	Yes	395	67.1	47	130/80
ADDITION (10), 2019	5.0	United Kingdom	Yes	Yes	336	59.5	56	130/80
J-DOIT3 (9), 2017	8.5	Japan	Yes	Yes	2,540	59.0	62	120/75
ACCORD (8), 2010	5.0	North America	No	No	4,733	62.2	52	120
SANDS (21), 2008	3.0	United States	No	Yes	499	56.0	34	115/75

BP indicates blood pressure.

Eight of the nine studies demonstrated a low risk of bias across all five domains, as assessed by RoB 2. One trial had some concerns regarding randomization because baseline blood pressure differed between groups (**Supplementary Figure 1**) (21).

### MACE

**Figure 2:**
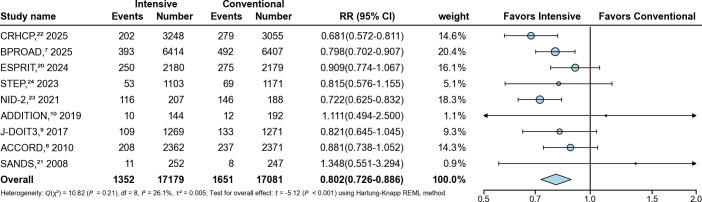
Risk of MACE with intensive versus conventional blood pressure control in patients with T2D. Data were pooled using a REML random-effects model with the Hartung-Knapp adjustment. CI indicates confidence interval; MACE, major adverse cardiovascular events; REML, restricted maximum likelihood; RR, risk ratio; and T2D, type 2 diabetes.

In the pooled analysis of 9 trials comprising 34,260 participants, MACE occurred in 1352 of 17,179 patients (7.9%) in the intensive blood pressure control group compared with 1651 of 17,081 patients (9.7%) in the conventional control group. Intensive control was associated with a significantly lower risk of MACE (RR, 0.80; 95% CI, 0.73–0.89; *P* < 0.001; **Figure 2**). No significant heterogeneity was observed across the included studies (*I*^2^ = 26.1%; *P* = 0.21). Assessment of publication bias via funnel plot inspection showed a symmetrical distribution, and Egger’s test was not statistically significant (*t* = 1.24; *P* = 0.25; **Supplementary Figure 2**). Furthermore, TSA demonstrated that the cumulative Z-curve crossed both the conventional significance boundary and the trial sequential monitoring boundary for benefit (**Supplementary Figure 3**). Results remained robust in the leave-one-out sensitivity analysis, with the pooled RR ranging from 0.78 to 0.82 (**Supplementary Figure 4**).

### Subgroup analyses

**Figure 3:**
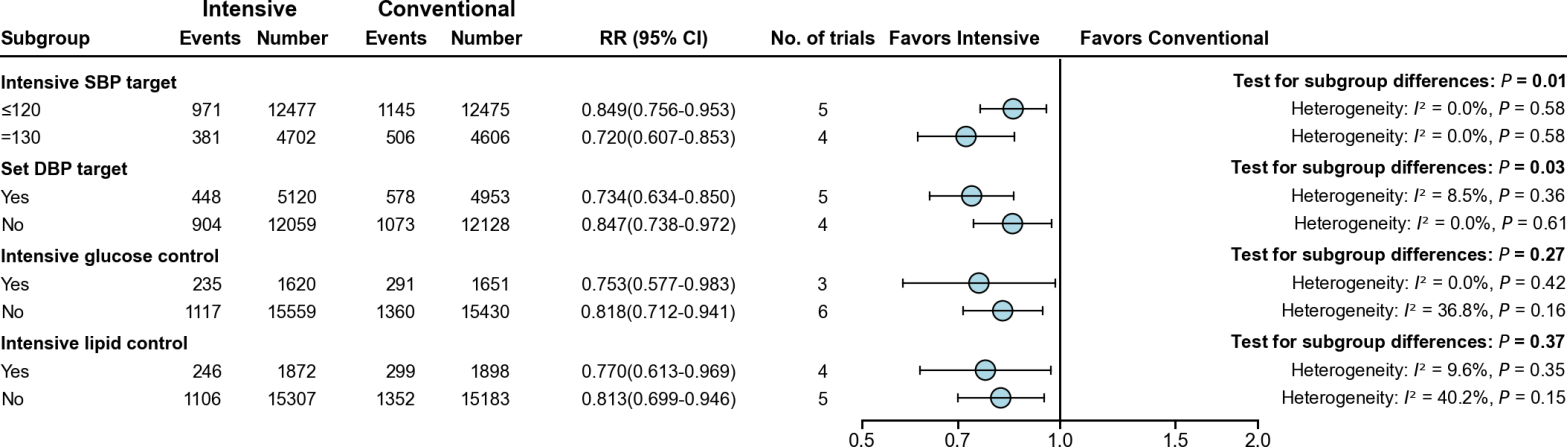
Subgroup analyses of the effect of intensive blood pressure control on MACE. Stratified analyses were performed by SBP target, concomitant DBP target, and intensive glucose or lipid control. CI indicates confidence interval; DBP, diastolic blood pressure; MACE, major adverse cardiovascular events; RR, risk ratio; SBP, systolic blood pressure; and T2D, type 2 diabetes.

Subgroup analyses stratified by blood pressure targets indicated a significant interaction between the treatment effect and target levels (*P* for interaction = 0.01; **Figure 3**). The magnitude of risk reduction appeared more pronounced in trials aiming for an SBP target of <130 mm Hg (RR, 0.72; 95% CI, 0.61–0.85) compared with those targeting ≤120 mm Hg (RR, 0.85; 95% CI, 0.76–0.95). Additionally, protocols incorporating a DBP target were associated with greater benefit (RR, 0.73; 95% CI, 0.63–0.85) than those focusing solely on SBP targets (RR, 0.85; 95% CI, 0.74–0.97; *P* for interaction = 0.03). Conversely, no significant interactions were observed regarding concomitant metabolic management; treatment benefits were consistent regardless of intensive glucose control [RR, 0.75 (95% CI, 0.58–0.98) vs RR, 0.82 (95% CI, 0.71–0.94); *P* for interaction = 0.27] or lipid control [RR, 0.77 (95% CI, 0.61–0.97) vs RR, 0.81 (95% CI, 0.70–0.95); *P* for interaction = 0.37].

### Components of MACE

**Figure 4:**
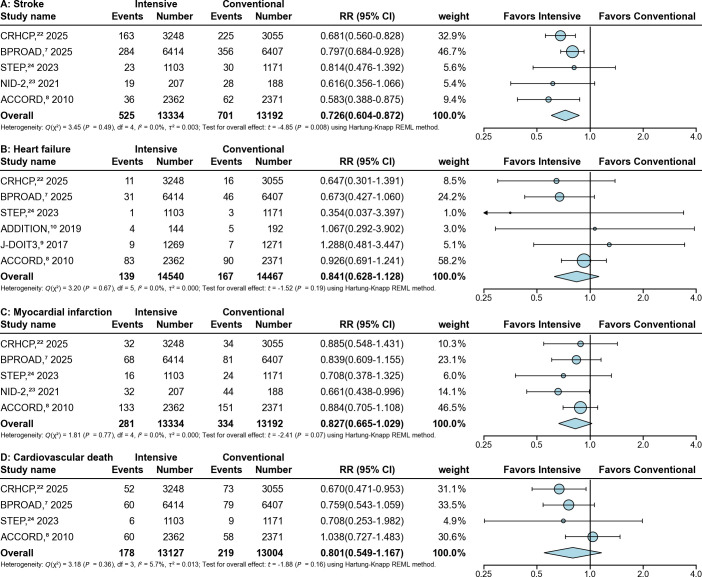
Risk of individual components of MACE. Forest plots show the pooled RRs for **(A)** stroke, **(B)** heart failure, **(C)** myocardial infarction, and **(D)** cardiovascular death using a REML random-effects model with the Hartung-Knapp adjustment. CI indicates confidence interval; MACE, major adverse cardiovascular events; REML, restricted maximum likelihood; and RR, risk ratio.

Regarding individual components of MACE, intensive treatment was associated with a significantly lower risk of stroke (5 studies), which occurred in 525 of 13,334 participants (3.9%) in the intensive group versus 701 of 13,192 participants (5.3%) in the conventional group (RR, 0.73; 95% CI, 0.60–0.87; *P* = 0.008; **Figure 4A**). However, no significant associations were observed for other components. The risk of heart failure (6 studies) did not differ significantly between groups (1.0% vs 1.2%; RR, 0.84; 95% CI, 0.63–1.13; *P* = 0.19; **Figure 4B**). Similarly, there were no significant reductions associated with intensive treatment for myocardial infarction (2.1% vs 2.5%; RR, 0.83; 95% CI, 0.67–1.03; *P* = 0.07; **Figure 4C**) or cardiovascular death (1.4% vs 1.7%; RR, 0.80; 95% CI, 0.55–1.17; *P* = 0.16; **Figure 4D**).

### Adverse events

**Figure 5:**
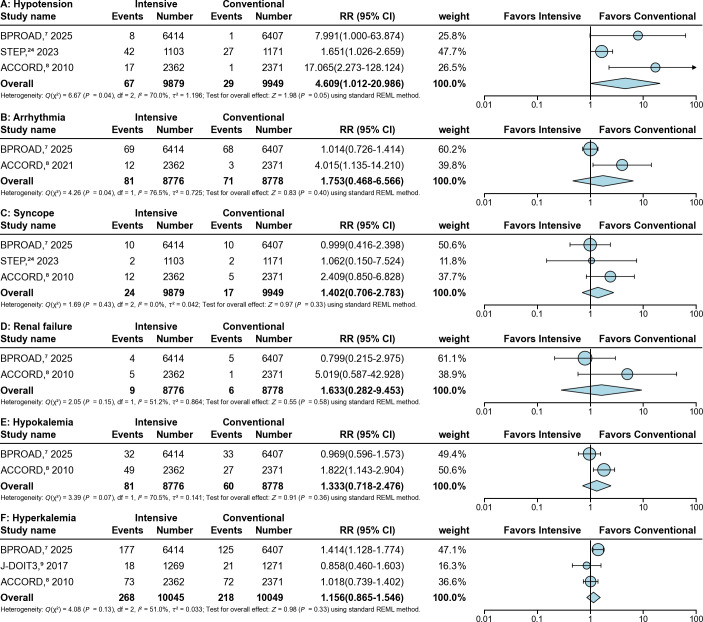
Risk of adverse events with intensive blood pressure control. Forest plots display the RRs of **(A)** hypotension, **(B)** arrhythmia, **(C)** syncope, **(D)** renal failure, **(E)** hypokalemia, and **(F)** hyperkalemia. Data were synthesized using a standard REML random-effects model due to the limited number of studies. CI indicates confidence interval; REML, restricted maximum likelihood; and RR, risk ratio.

Analysis of safety outcomes revealed a borderline increase in the risk of hypotension in the intensive treatment group. Hypotension (3 studies) was reported in 67 of 9879 participants (0.7%) in the intensive group compared with 29 of 9949 participants (0.3%) in the conventional group (RR, 4.61; 95% CI, 1.01–20.99; *P* = 0.05; **Figure 5A**). No significant differences were found for other adverse events, including arrhythmia (RR, 1.75; 95% CI, 0.47–6.57; *P* = 0.40; **Figure 5B**), syncope (RR, 1.40; 95% CI, 0.71–2.78; *P* = 0.33; **Figure 5C**), renal failure (RR, 1.63; 95% CI, 0.28–9.45; *P* = 0.58; **Figure 5D**), hypokalemia (RR, 1.33; 95% CI, 0.72–2.48; *P* = 0.36; **Figure 5E**), or hyperkalemia (RR, 1.16; 95% CI, 0.87–1.55; *P* = 0.33; **Figure 5F**). Sensitivity analyses using the Mantel-Haenszel method for these sparse-event data showed the similar results (**Supplementary Figure 5**).

## Discussion

This meta-analysis revealed that intensive blood pressure control was associated with a significant reduction in the risk of MACE in patients with T2D. These findings were robust and characterized by minimal heterogeneity, negligible publication bias, and consistent benefits across individual MACE components. However, among the included studies, only the BPROAD, NID-2, and CRHCP trials achieved statistical significance, which may partly reflect longer follow-up periods and greater event accrual (7, 22, 23, 25). The lack of statistical significance in the ACCORD trial may be attributed to a lower-than-expected event rate, which compromised the statistical power (8, 26). Similarly, data from ESPRIT and STEP were derived from subgroups of general hypertensive populations, resulting in fewer events and wider confidence intervals (20, 24, 27). Conversely, point estimates from ADDITION and SANDS favor conventional control. These discrepancies likely reflected statistical imprecision driven by extremely low event counts (<30 total events) (10, 21). This limitation stemmed from inherent differences in study design: ADDITION enrolled patients with newly diagnosed T2D and low baseline cardiovascular risk, whereas SANDS was primarily powered to assess carotid intima–media thickness rather than MACE outcomes (10, 21).

Previous guidelines associated lower SBP targets with reduced cardiovascular risk, citing a log-linear relationship observed down to 115 mm Hg (4, 5, 28–33). Consequently, it was hypothesized that a target of 120 mm Hg would yield superior outcomes compared with one of 130 mm Hg. However, the subgroup analysis shown in **Figure 3** indicated that trials targeting 130 mm Hg were associated with a greater reduction in cardiovascular risk than those targeting 120 mm Hg. This apparent discrepancy likely stems from methodological heterogeneity rather than a genuine lack of benefit from the lower target. The lower RR observed in the subgroup targeting 130 mm Hg was primarily driven by the CRHCP and NID-2 trials (22, 23). In the CRHCP trial, the achieved SBP in the conventional group was 147.7 mm Hg, significantly exceeding that reported in other included studies (**Supplementary Table 3**) (22). This suboptimal control in the conventional arm resulted in a higher event rate, thereby amplifying the relative benefit of the intensive intervention (22). Additionally, the NID-2 trial implemented simultaneous intensive control of lipids, glucose, and blood pressure. Given the follow-up exceeding 10 years, the observed cardiovascular benefits may not be solely attributable to blood pressure lowering (23). Moreover, we note that J-DOIT3, the only RCT directly comparing SBP targets of 120 mm Hg and 130 mm Hg, showed a numerically lower risk of MACE (RR, 0.821; 95% CI, 0.645-1.045), though no statistical significance was observed (9). It raises the possibility that a blood pressure target of 120 mm Hg may confer greater benefit in MACE, compared to 130 mm Hg. Therefore, more similar studies are needed to determine the optimal SBP target.

The necessity of establishing a concurrent DBP target alongside an intensive SBP goal remains debated. The HOT trial suggested that lowering DBP below 80 mm Hg is associated with reduced cardiovascular risk (34). However, concerns regarding the “J-curve” phenomenon have emerged, suggesting that excessive DBP reduction may compromise coronary perfusion (35–39). Trials such as ACCORD, SPRINT, STEP, ESPRIT and BPROAD prioritized SBP targets without specific DBP goals (6–8, 20, 24, 39). This design may reflect study objectives and safety considerations, acknowledging that lowering SBP inevitably reduces DBP. Interestingly, our subgroup analysis indicated that strategies incorporating a specific DBP target were associated with greater benefits. This finding was primarily driven by the CRHCP trial (22, 25). CRHCP also achieved the largest between-group separation in achieved blood pressure among included studies (22, 25). Unlike studies in academic centers, the CRHCP trial was conducted in rural communities with less stringent monitoring (22, 25). In such pragmatic settings, explicitly targeting both SBP and DBP may reinforce treatment adherence. Therefore, DBP should be monitored when implementing intensive SBP lowering strategies.

Subgroup analysis revealed that, compared with intensive blood pressure control alone, concomitant intensive glucose control offered modest cardiovascular benefits. This finding aligns with trials focusing on intensive glucose control, such as ADVANCE and ACCORD, which indicated that such control offers only modest cardiovascular protection (RR 0.90–0.95) and was notably more effective in reducing the risk of microvascular complications (RR 0.80–0.90), including diabetic nephropathy and retinopathy (40–42). Regarding intensive lipid control, although it is well-established that lipid lowering reduces MACE incidence, this subgroup analysis observed limited additive cardiovascular benefits from simultaneous intensive lipid-lowering therapy (43–45). This may be attributed to the slow regression of atherosclerosis. The follow-up periods in the included studies were typically 3 to 5 years, which might have been insufficient to fully capture the incremental cardiovascular benefits of intensive lipid modulation in addition to blood pressure control (7–10, 20–22, 24–26). Therefore, a comprehensive approach targeting glucose and lipids alongside blood pressure remains clinically justified for broad organ protection, despite the lack of evident synergistic macrovascular effects in this analysis.

Safety is an important consideration when pursuing lower blood pressure targets (46–48). This meta-analysis indicated that intensive blood pressure control, while correlating with cardiovascular protection, was associated with an elevated risk of adverse events, particularly hypotension. Notably, the incidence of adverse events was significantly lower in studies published in 2025 than in trials published before 2010 (7, 8). This improvement was attributed primarily to the evolution of pharmacological strategies, including the use of newer antihypertensive agents with better safety profiles and the adoption of low-dose combination therapies, which minimized dose-dependent adverse events while maintaining efficacy (7, 8, 49–54). Therefore, when intensive blood pressure control is implemented, the prioritization of newer agents with superior tolerability and the use of combination strategies is advised to maximize cardiovascular benefits while minimizing safety risks.

Although the GRADE assessment indicated moderate certainty of evidence for our primary outcome, as detailed in **Supplementary Table 4**, this meta-analysis has several limitations. First, three of the included studies were based on *post hoc* subgroup analyses of broader hypertension trials. Participants in these studies generally had higher baseline blood pressure and higher cardiovascular risk, which may overestimate the benefits of intensive intervention in the general T2D population. Second, recent studies generally use a broader definition of MACE than earlier studies such as the ACCORD trial (**Supplementary Table 5**), which may make the observed effect more pronounced. Third, four included studies were conducted in China and accounted for approximately 56.2% of the overall weight, which may limit the generalizability of these findings to other ethnic groups. Fourth, all included trials employed an open-label design, which may lead to higher reported rates of adverse events such as hypotension. Fifth, some studies did not clearly specify the method of blood pressure measurement. If unattended measurements were used, the resulting blood pressure readings may have been lower. Therefore, RCTs with larger sample sizes and longer follow-up periods, more comprehensive outcome measures (especially focusing on adverse events) and more rigorous methodology are still needed.

## Conclusions

Intensive blood pressure control significantly reduces the risk of MACE in patients with T2D. Strategies incorporating explicit DBP targets may offer substantial benefits. However, it should be paid attention to monitoring and preventing adverse events.

## Data Availability

The original contributions presented in the study are included in the article/**Supplementary Material**. Further inquiries can be directed to the corresponding author.
