# Supplementary material for: Intensive blood pressure control in type 2 diabetes: a meta-analysis of randomized controlled trials
Source: Front Endocrinol (Lausanne). 2026 Jun 15;17:1850865. doi: 10.3389/fendo.2026.1850865 (PMC13310730; doi:10.3389/fendo.2026.1850865)
Supplement: Supplementary file 1 [file SupplementaryFile1.docx]

Supplementary Material

# Supplementary Figures

**
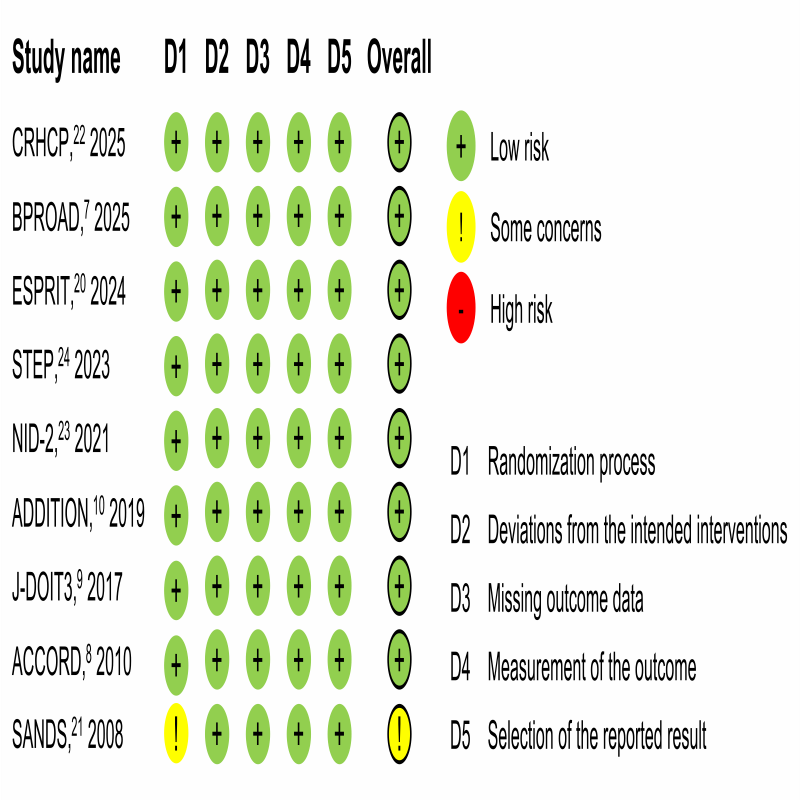
**

**Supplementary Figure 1. Risk of bias assessment.** Risk of bias summary for included studies assessed using the RoB 2 tool. RoB 2 indicates Revised tool to assess risk of bias in randomized trials.


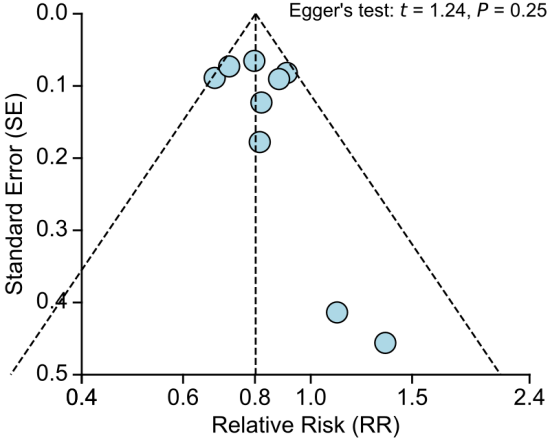


**Supplementary Figure 2. Funnel plot for the assessment of publication bias for MACE.** MACE indicates major adverse cardiovascular events.


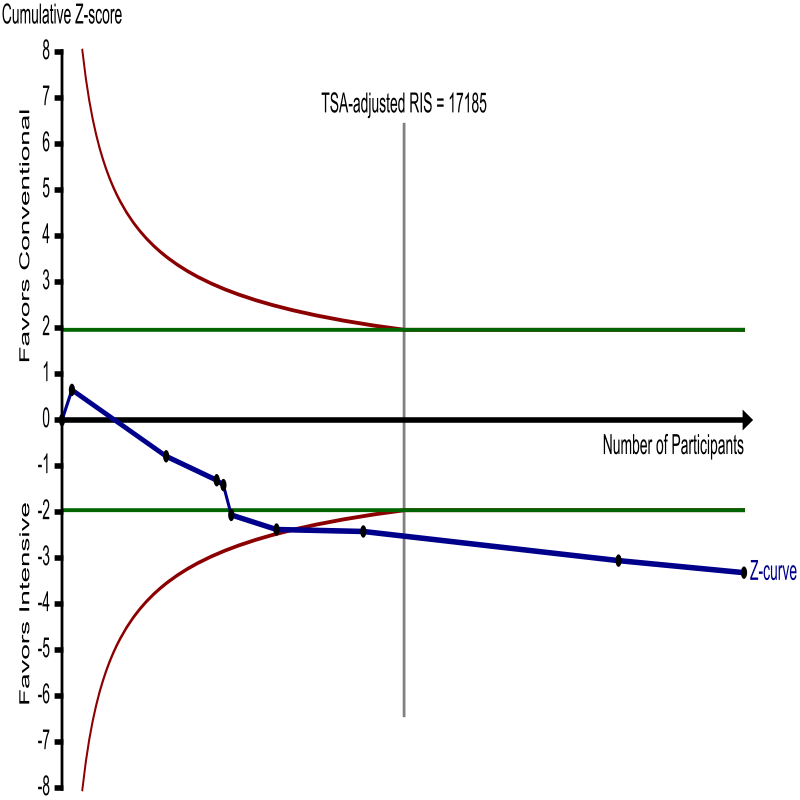


**Supplementary Figure 3. Trial sequential analysis for MACE.** MACE indicates major adverse cardiovascular events; and RIS, required information size.


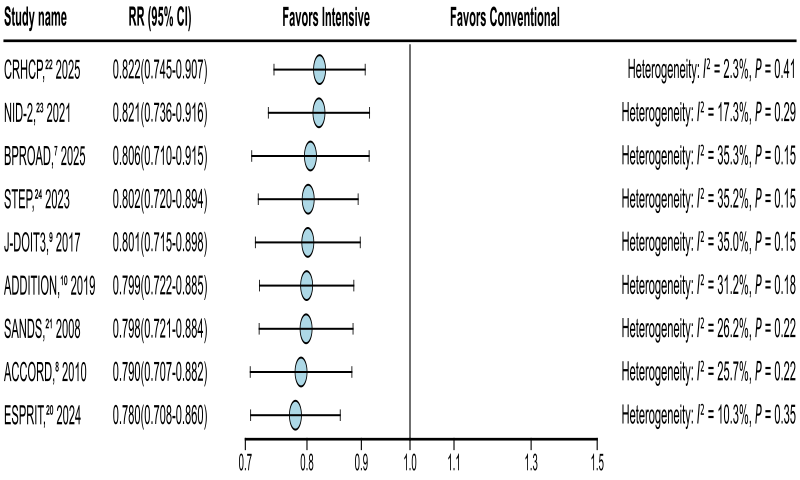


**Supplementary Figure 4. Leave-one-out sensitivity analysis for MACE.** CI indicates confidence interval; MACE, major adverse cardiovascular events; and RR, risk ratio.


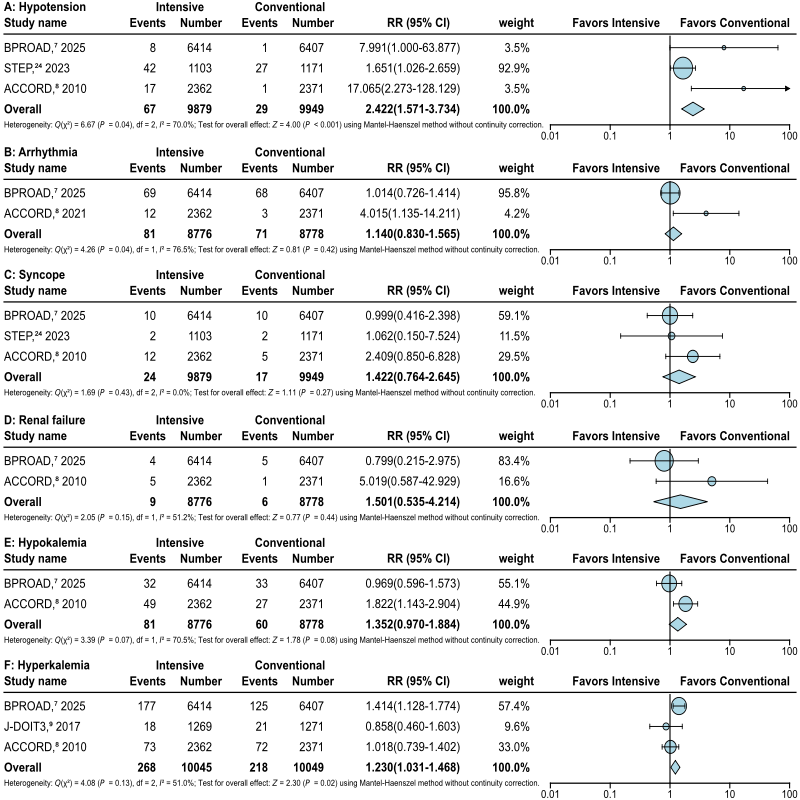


**Supplementary Figure 5. Sensitivity analysis of adverse event risk with intensive blood pressure control.** Forest plots display the RRs of (A) hypotension, (B) arrhythmia, (C) syncope, (D) renal failure, (E) hypokalemia, and (F) hyperkalemia. Data were synthesized using the Mantel–Haenszel method without applying a continuity correction to zero-event cells. CI indicates confidence interval; RR, risk ratio.

**Supplementary Tables**

Supplementary Table 1. PRISMA 2020 Checklist.


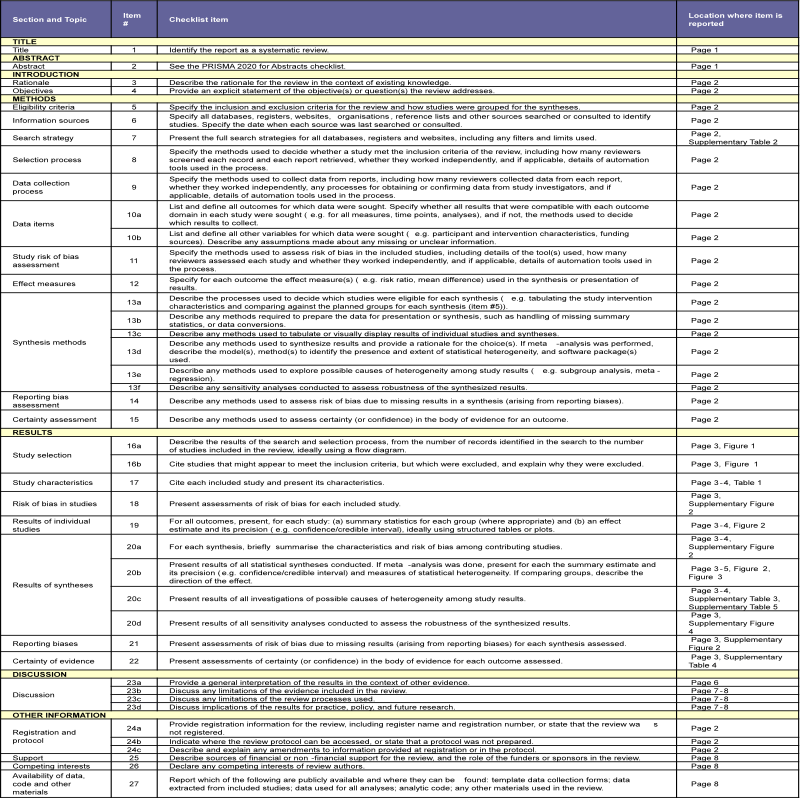


Supplementary Table 2. Search Strategy for Electronic Databases.


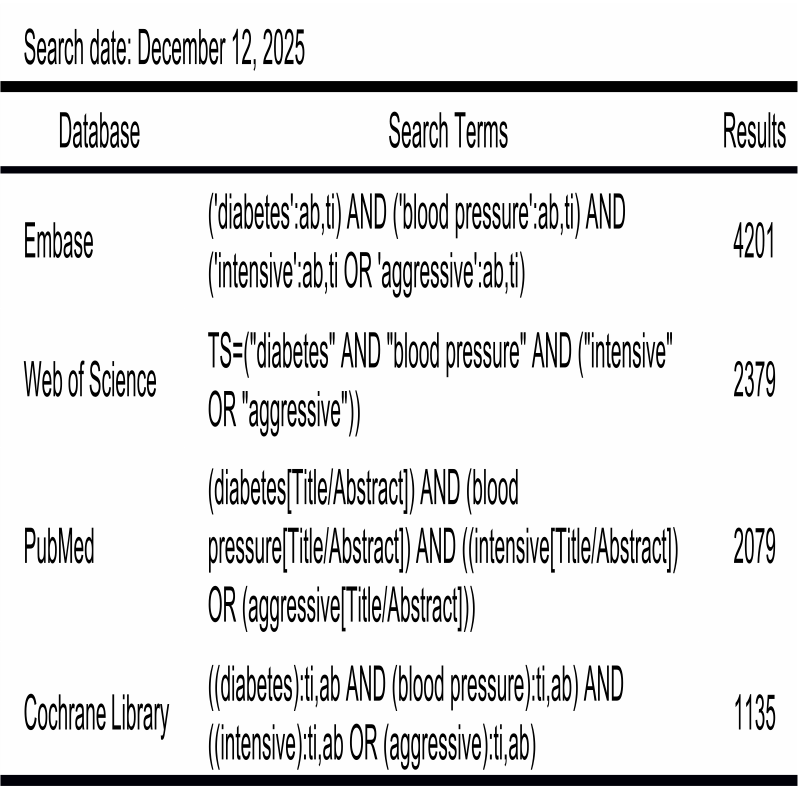


Supplementary Table 3. Baseline and Achieved BP Characteristics of Included Studies.


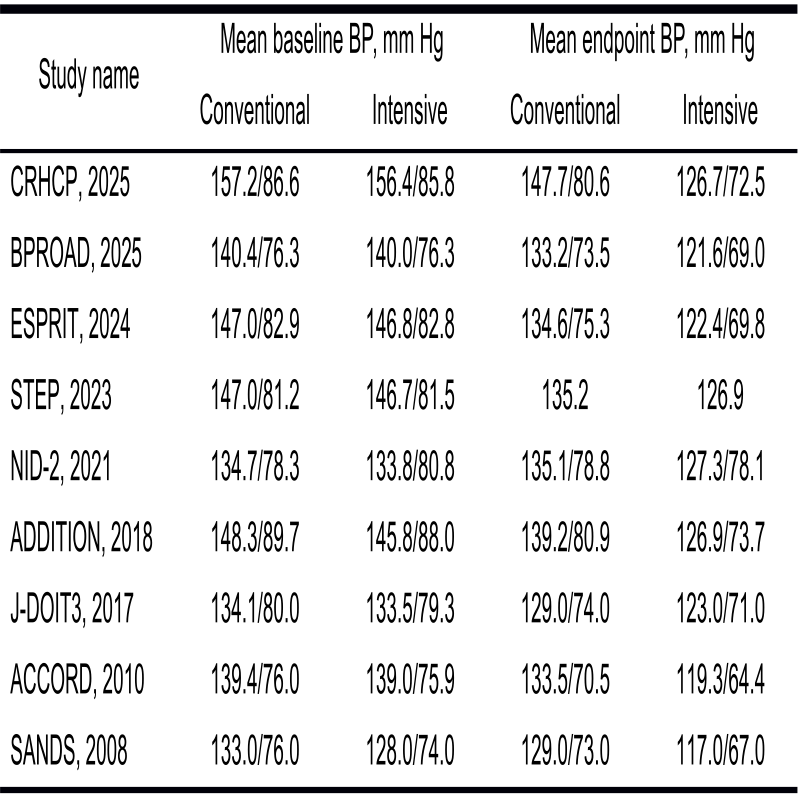


BP indicates blood pressure.

Supplementary Table 4. GRADE Summary of Findings for MACE.

| Outcome and follow-up | Patients (studies), N | Relative effect (95% CI) | **Absolute effects (95% CI)** | | | Certainty | What happens |
| --- | --- | --- | --- | --- | --- | --- | --- |
|  |  |  | **Conventional** | **Intensive** | **Difference** |  |  |
| MACE, Follow-up duration: 3-10.5 years | 34260 (9 RCTs) | **RR = 0.802** (0.726 to 0.886) | 97 per 1,000 | **78 per 1,000** (70 to 86) | **19 fewer per 1,000** (from 26 fewer to 11 fewer) | ⨁⨁⨁◯ Moderate | For patients with type 2 diabetes, intensive blood pressure control probably reduces the risk of MACE compared with conventional blood pressure control (RR=0.802, 95% CI 0.726–0.886; moderate-certainty evidence). The certainty was downgraded because of indirectness related to inclusion of diabetic subgroups from broader hypertension trials, methodological concerns in some studies, and clinical heterogeneity in intervention strategies. |

CI indicates confidence interval; MACE, major adverse cardiovascular events; and RR, risk ratio.

Supplementary Table 5. MACE definitions across included trials.

| Study name | Components of MACE |
| --- | --- |
| CRHCP, 2025 | myocardial infarction, stroke, heart failure, and cardiovascular death |
| BPROAD, 2025 | myocardial infarction, stroke, heart failure, and cardiovascular death |
| ESPRIT, 2024 | myocardial infarction, stroke, heart failure, coronary or non-coronary revascularization, and cardiovascular death |
| STEP, 2023 | myocardial infarction, stroke, heart failure, hospitalization for unstable angina, coronary revascularization, atrial fibrillation, and cardiovascular death |
| NID-2, 2021 | myocardial infarction, stroke, coronary revascularization, major lower-limb amputation, and cardiovascular death |
| ADDITION, 2018 | myocardial infarction, stroke, coronary revascularization, peripheral vascular angioplasty, and carotid endarterectomy |
| J-DOIT3, 2017 | myocardial infarction, stroke, coronary revascularization, carotid or cerebral revascularization, and all-cause mortality |
| ACCORD, 2010 | myocardial infarction, stroke, and cardiovascular death |
| SANDS, 2008 | myocardial infarction, stroke, unstable angina, coronary revascularization, carotid revascularization, and cardiovascular death |

MACE indicates major adverse cardiovascular events.
